# Relapsing autoimmune inner ear disease with significant response to methotrexate and azathioprine combination therapy: A case report and mini literature review

**DOI:** 10.1097/MD.0000000000033889

**Published:** 2023-06-09

**Authors:** Kuan-Hsuan Huang, Hsiao-Ching Lin, Chia-Der Lin, Po-Chang Wu

**Affiliations:** a Department of Education, Shin Kong Wu Ho Su Memorial Hospital, Taipei, Taiwan; b Department of Education, Taichung Veterans General Hospital, Taichung, Taiwan; c School of Medicine, College of Medicine, China Medical University, Taichung, Taiwan; d Department of Otolaryngology Head and Neck Surgery, China Medical University Hospital, Taichung, Taiwan; e Rheumatology and Immunology Center, China Medical University Hospital, Taichung, Taiwan.

**Keywords:** autoimmune inner ear disease, azathioprine, immunosuppressant therapy, methotrexate, pure-tone

## Abstract

Autoimmune inner ear disease typically presents with bilateral hearing loss that progresses over weeks or months though its mechanisms are unknown. Corticosteroids are the first-line treatment, but their responses are variable and relapses are frequent. Thus, many experts have sought to replace corticosteroids with immunosuppressive agents.

**Patient concerns:**

A 35-year-old woman experienced a progressive hearing impairment, initially on the left side and later becoming bilateral. Her response to corticosteroid monotherapy was temporary, and there have been two relapse episodes over several months.

**Diagnoses:**

Autoimmune inner ear disease was considered due to evidence of autoimmunity combined with a clinical course of bilateral and recurrent sensorineural hearing loss and a partial response to corticosteroid therapy.

**Interventions:**

The patient received a 3-day mini-pulse of methylprednisolone at 250 mg/d, followed by 12 mg/d maintenance, and concurrently began an azathioprine regimen gradually increasing to 100 mg/day as a corticosteroid-sparing agent.

**Outcomes:**

Three weeks after immunosuppressive therapy, hearing and pure-tone audiometry improved, and after 7 weeks, methylprednisolone was tapered to 8 mg/d. The dosage was further reduced by adding methotrexate at 7.5 mg/week, resulting in a reduction to 4 mg/d as maintenance therapy after 4 weeks.

**Lessons:**

For patients who are unresponsive to corticosteroids or experience difficulty tolerating them, a combination therapy of methotrexate and azathioprine is recommended as a viable alternative as this regimen is well-tolerated and yields positive outcomes.

## 1. Introduction

Autoimmune inner ear disease (AIED) typically presents with bilateral hearing loss that progresses over weeks or months, but the mechanisms underlying AIED remain unknown. Corticosteroids (e.g., oral prednisone, intratympanic methylprednisolone, intratympanic dexamethasone) are considered the first-line treatment; however, treatment responses are variable. Besides, many patients experience frequent relapses when using corticosteroid monotherapy. Due to the above reasons, many experts tried to use immunosuppressive agents to replace the use of corticosteroids. This article reports a woman with AIED, whose response to corticosteroid monotherapy was transient, accompanied by 2 relapse episodes over months. We prescribed methotrexate and azathioprine as combination therapy, which led to a significant improvement in her pure-tone average. We recorded all pure-tone audiometry (PTA) data to evaluate the response to treatment. In addition, we reviewed the literature to assess the effects of current immunosuppressant therapy options for improving AIED.

## 2. Case description

**Figure 1.**
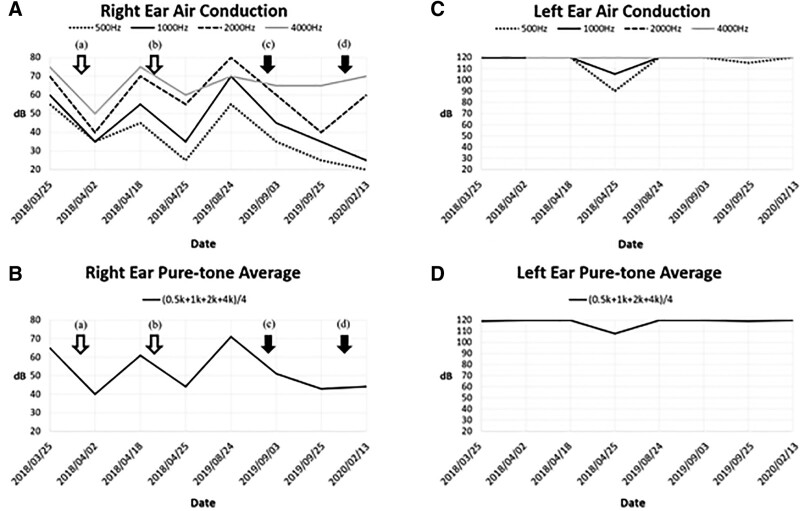
Pure-tone audiometry for both ears by air conduction at 500, 1000, 2000, and 4000 Hz. The X-axis indicates the time point at which pure-tone audiometry was performed. The Y-axis indicates the lowest decibel (dB) the patient was able to hear. The arrows indicate the timing of treatments, with empty arrows representing steroids alone and solid arrows representing treatment with steroids combined with a nonsteroidal immunosuppressant. (A) Pure-tone audiometry and (B) pure-tone average of the right ear. (A) Treatment initiated on March 26, 2018, consisting of intratympanic corticosteroid injection therapy (5 mg dexamethasone) and oral corticosteroids (60 mg/d prednisolone, tapered to 20 mg/d starting April 2, 2018). (B) Treatment initiated on April 19, 2018, consisting of intratympanic corticosteroid injection therapy (5 mg dexamethasone) and oral corticosteroids (60 mg/d prednisolone, tapered to 15 mg/d starting April 26, 2018). (C) Treatment initiated on August 24, 2019, consisting of 250 mg/d of mini-pulse methylprednisolone for 3 days (September 3–September 5), oral corticosteroids (12 mg/d methylprednisolone, tapered to 8 mg/d starting October 24, 2019), 100 mg/d azathioprine, 400 mg/d hydroxychloroquine, and 100 mg/d aspirin 100 mg/day as adjuvant therapy. (D) Treatment initiated on December 19, 2019, consisting of oral corticosteroids (8 mg/d methylprednisolone, tapered to 4 mg/d starting January 10, 2020), 100 mg/d azathioprine, 400 mg/d hydroxychloroquine, and 7.5 mg/week methotrexate. (C) Pure-tone audiometry and (D) pure-tone average of the left ear. Left ear air conduction was poor since her first episode during pregnancy in October 2014. Without timely medical treatment, her left ear hearing function deteriorated gradually. No significant improvement was noted in left hearing loss even after right hearing loss improved.

**Figure 2.**
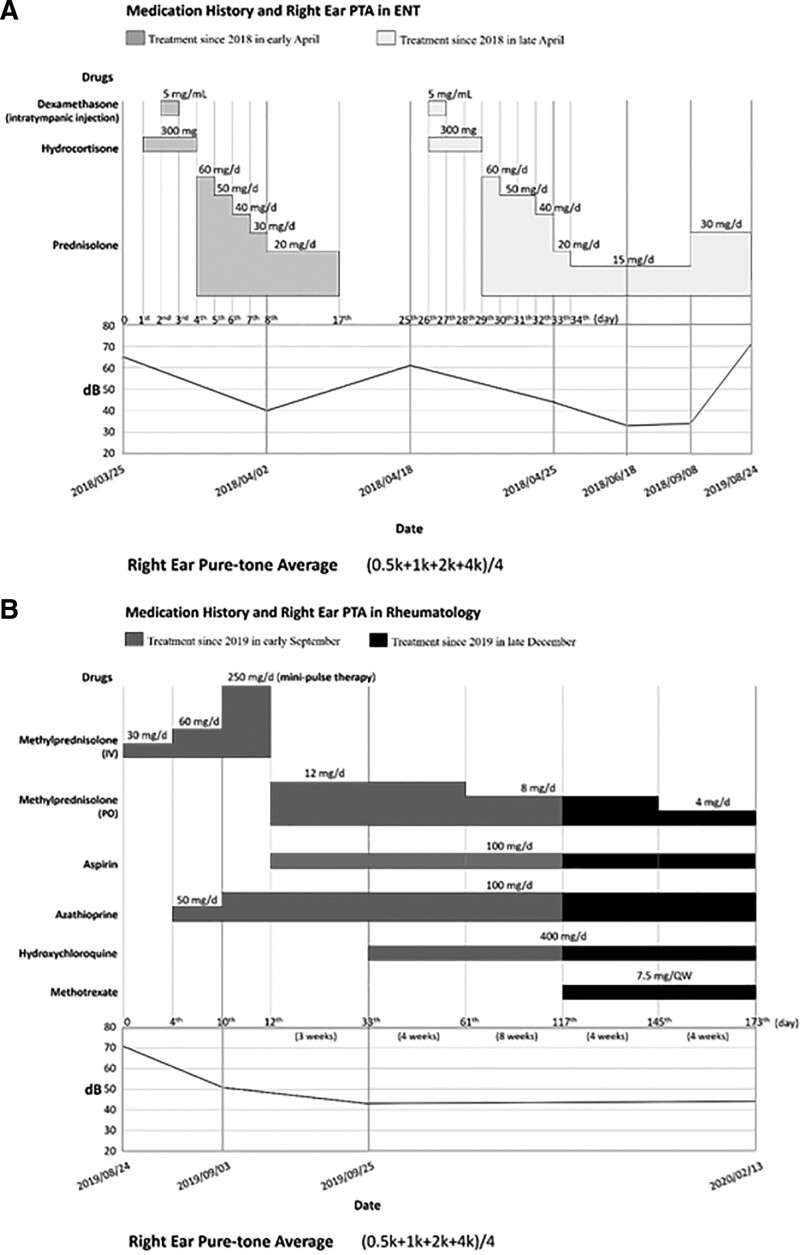
Pure-tone average of the right ear. (A) Medical treatment in the ENT (Otolaryngology) Department in April. Treatment began on March 26, 2018, with an intratympanic injection of dexamethasone at 5 mg/mL in the right ear and 3 days of intravenous hydrocortisone 300 mg/d, then reduced to 60 mg/d of oral prednisolone. Corticosteroid therapy only partially improved her hearing (from 65 to 40 dB), which declined again after 16 days (from 40 to 60 dB). A second course of treatment (intratympanic dexamethasone and systemic corticosteroid therapy) began on April 19 and improved her hearing (from 60 to 45 dB). Maintenance treatment with 15 mg/d of oral prednisolone started on April 26, 2018. (B) Medical treatment in the Rheumatology Department (from September 2019 to February 2020). In September 2019, the patient received a 3-day mini-pulse of methylprednisolone at 250 mg/d, followed by 12 mg/d maintenance, and concurrently began an azathioprine regimen gradually increasing to 100 mg/day as a corticosteroid-sparing agent. Aspirin (100 mg/d) and hydroxychloroquine (400 mg/d) were added due to weakly positive lupus anticoagulants in the patient’s blood. Three weeks after immunosuppressive therapy, hearing and PTA improved, and after 7 weeks, methylprednisolone was tapered to 8 mg/d. The dosage was further reduced by adding methotrexate at 7.5 mg/week, resulting in a reduction to 4 mg/d as maintenance therapy after 4 weeks. PTA = pure-tone audiometry.

A 35-year-old woman experienced progressive left-sided hearing impairment, which started in October 2014. At the time of initial symptom presentation, the patient was pregnant at 16 weeks of gestation and refused to accept medical treatment after seeking medical attention due to fears that the medication posed a pregnancy risk. Her left hearing function deteriorated gradually until she reported profound left-sided hearing loss at 31 weeks of gestation. She delivered a healthy full-term baby in April 2015. Due to her concern about the potential adverse effects of drug treatment on breastfeeding infants and her preservation of normal hearing in her right ear at that time, she did not seek any treatment postpartum until progressive right-side hearing impairment developed starting in March 2018 (Fig. 1). A physical examination revealed bilateral intact eardrums, positive Rinne test in both ears and no obvious sound lateralization in Weber test due to both ear hearing loss. Also, no joint tenderness, swelling, deformity, or motion limitation was observed. No erythema, petechiae, blisters, objective pulsatile tinnitus, traumatic scar, or focal neurologic signs were identified. Magnetic resonance imaging of the internal auditory canal revealed no lesions in the paranasal sinuses, mastoids, ventricular system, or brain parenchyma. She received treatment starting on March 26, 2018, which included an intratympanic dexamethasone injection at 5 mg/mL in her right ear and 3 days of intravenous hydrocortisone at 300 mg/d. Then, the dosage was reduced to oral prednisolone at 60 mg/d. The corticosteroid dosage was gradually tapered daily and eventually reduced to 20 mg/d of prednisolone (Fig. 2A). However, corticosteroid therapy only partially improved hearing function (from 65 to 40 dB). Moreover, her hearing deteriorated again after 16 days (from 40 to 60 dB) (Fig. 1), so she received a second course of treatment with intratympanic dexamethasone and systemic corticosteroid therapy starting on April 19, 2018. After this course of treatment, her hearing improved again (from 60 to 45 dB) (Fig. 1). Therefore, she started maintenance treatment with oral prednisolone at 15 mg/d starting on April 26, 2018 (Fig. 2A). Over the next 4 months, she experienced many side effects of corticosteroids, including moon face, weight gain, acne, easy bruising, and intermittent urinary tract infections. However, because her hearing did not worsen during this period, she did not actively seek medical care or continued monitoring after September 8, 2018. Nevertheless, she reported profound right-sided hearing loss in late June 2019, and oral prednisolone at 15 mg/d was resumed. When she showed no significant improvement, she was referred to a rheumatology clinic in August 2019.

On August 24, 2019, a PTA test indicated a severe sensorineural hearing impairment, with a baseline of 71 dB in the right ear and 120 dB in the left ear (Fig. 1). Serology tests were unremarkable, except for borderline positive lupus anticoagulant and antithyroid-stimulating hormone receptor antibody (Table 1). A diagnosis of systemic lupus erythematosus (SLE) was excluded due to the patient’s negative antinuclear antibody test and lack of clinical manifestations associated with SLE, such as skin rash, arthritis, cytopenia, nephritis, proteinuria, and other SLE-specific autoantibodies. A diagnosis of autoimmune thyroiditis was also excluded due to the lack of any clinical symptoms or signs of hyperthyroidism. AIED was considered due to evidence of autoimmunity combined with a clinical course of bilateral and recurrent sensorineural hearing loss and a partial response to corticosteroid therapy (Tables 2 and 3). The patient was administered 250 mg/d of mini-pulse methylprednisolone for 3 days in September 2019, followed by 12 mg/d of methylprednisolone. We also administered azathioprine and gradually increased the dosage to 100 mg/d to serve as a corticosteroid-sparing agent. While the patient had no history of thrombotic events or pregnancy-related complications, which do not fulfill the diagnosis of antiphospholipid syndrome, the presence of weakly positive lupus anticoagulants in their blood led us to administer 100 mg/d of aspirin and 400 mg/d of hydroxychloroquine as adjuvant therapy (Fig. 2B). Three weeks after the initiation of immunosuppressive therapy, the patient’s hearing function improved significantly (Fig. 1), and a follow-up PTA also showed improvement. Seven weeks after starting immunosuppressive therapy, the methylprednisolone dosage was tapered to 8 mg/d (Fig. 2B). Due to the patient’s prior experience of enduring many side effects of corticosteroids, to reduce the methylprednisolone dosage even further, we added methotrexate at 7.5 mg/week as a corticosteroid-sparing agent with a full azathioprine dose (100 mg/d). After 4 weeks of administering methotrexate at 7.5 mg/week, the patient’s methylprednisolone was reduced to 4 mg/d as maintenance therapy (Fig. 2B).

**Table 1:** Laboratory data in August 2019.

	Reference range	2019/08		Reference range	2019/08
**ANA**	Negative	Negative	**Lupus anticoagulant**	Normalize Ratio < 1.2	1.3* (weakly present)
**Anti-SM antibody**	Negative	Negative	**Anticardiolipin IgM**	Negative	Negative
**Anti-RNP antibody**	Negative	Negative	**Anticardiolipin IgG**	Negative	Negative
**Anti-SS-A/SS-B antibody**	Negative	Negative	**Anti-beta2 glycoprotein IgM**	Negative	Negative
**Anti-dsDNA antibody**	Negative	Negative	**Anti-beta2 glycoprotein IgG**	Negative	Negative
**ANCA**	Negative	Negative	**Rheumatoid Factor**	Negative	Negative
**TSH receptor antibody**	<15%	17.2* (weakly positive)	**IgG (mg/dL**)	751–1560	1450 (normal)
**Antithyroglobulin antibody**	Negative	Negative	**IgA (mg/dL**)	82–453	343 (normal)
**Anti-TPO antibody**	Negative	Negative	**IgM (mg/dL**)	46–304	189 (normal)
**TSH (uIU/mL**)	0.34–5.6	2.287 (normal)	**C3 (mg/dL**)	79–152	163
**Free-T4 (ng/dL**)	0.54–1.40	1.00 (normal)	**C4 (mg/dL**)	16–38	49.0

ANA = antinuclear antibody, ANCA = antineutrophil cytoplasmic antibody, TPO = thyroid peroxidase, TSH = thyroid stimulating hormone.

* Higher than the normal range.

**Table 2:** Common differential diagnosis and clinical presentations of sensorineural hearing loss.

	Laterality	Clinical manifestations
Autoimmune disease	Usually bilateral and asymmetric	This condition may have a fluctuating or progressive clinical course, and its symptoms may be localized to the ear or indicative of systemic autoimmune disease.
Vascular disease	Unilateral	This condition typically presents with sudden onset and may be accompanied by other neurological signs or symptoms such as diplopia, nystagmus, facial asymmetry, limb clumsiness, or ataxia.
Infections	Unilateral or bilateral	This condition typically presents with sudden onset and may be accompanied by symptoms such as vertigo, facial pain, or paralysis.
Neoplasms	Unilateral or asymmetric	In addition to its primary symptoms, this condition may also be accompanied by other manifestations such as unilateral tinnitus, disequilibrium, dizziness, or headaches.

**Table 3:** Diagnosis approach and treatment.

Symptom	• Bilateral but asymmetric sensory neural hearing loss • Fluctuating or progressive hearing loss over weeks or months • Sometimes accompanied by functional vestibular impairment
Diagnosis	• Exclude other structural problems and infection • Clinical diagnosis • Good response to corticosteroid • Autoimmune titer may positive
Treatment	• Corticosteroid • Immunosuppressants, ex: cyclophospharnide, methotrexate, azathioprine • Biologic agents, ex: etanercept, infliximab, golimumab, adalimumab, rituximab, anakinra

## 3. Discussion

AIED is an uncommon disease that typically presents with bilateral hearing loss but is sometimes asymmetrical.^[1]^ The disease course progresses over weeks or months rather than days or years, unlike other hearing loss diseases, such as sudden deafness (occurs in minutes or hours), cochlear Meniere disease (sudden hearing loss with fluctuation), or cochlear (otosclerotic) deafness (progresses in years).^[2]^ Corticosteroids are the first-line treatment for AIE.^[2,3]^ Although about 70% of patients may initially respond to corticosteroid therapy, the treatment effect often decreases over time. Finally, only 14% of patients had real therapeutic responses to corticosteroids.^[4–8]^

McCabe first described the term AIED in 1979.^[2]^ The clinical features of AIED are variable and can include fluctuating or progressive hearing loss (over a period of weeks or months), sometimes accompanied by functional vestibular impairment.^[1]^ AIED can present as an isolated inner ear disease or as secondary to systemic autoimmune disorders.^[6,9,10]^ An AIED diagnosis depends on the clinical presentation and responsiveness to corticosteroid treatment,^[11]^ with no currently available serologic marker (Tables 2 and 3).^[12]^ Although many biomarkers have been reported to detect AIED, including myelin P0 protein,^[13]^ the 58-kDa protein,^[14]^ and the 68-kDa protein,^[15]^ they are all in the inner ear and limited to use as research tools due to low sensitivity.^[16]^ In this case, these biomarkers were not utilized to diagnose the patient.

The precise mechanisms underlying AIED have not yet been established in humans due to the lack of available histological tissue specimens. Most studies use an animal model to test hypotheses related to AIED.^[17,18]^ Recent studies have reported that immune-competent cells and macrophages found in the endolymphatic sac of the inner ear may represent the primary site of the immune response.^[1,18–22]^ Many clinical cases have demonstrated a relationship between progressive hearing loss and autoimmune diseases, suggesting that immunocomplexes may damage the inner ear tissue.^[20]^ Lymphocyte and immunoglobulins have also been shown to enter the ear and cause immune reactions in some animal studies, suggesting that both cell- and antibody-mediated immunity are involved in AIED pathogenesis.^[17,18,23]^

Corticosteroids remain the mainstay treatment for AIED.^[24]^ Intratympanic steroid injections have emerged as a viable alternative treatment with greater long-term tolerability.^[24–28]^ However, nonsteroidal immunosuppressants (cyclophosphamide, methotrexate, and azathioprine) and biological agents (TNF-α inhibitors, antiCD20 antibody, and IL-1 receptor antagonists) are potential options for refractory AIED.

Cyclophosphamide is no longer frequently used to treat AIED due to its side effect profile and poor results in hearing improvement.^[24,29–32]^

Methotrexate has been used as an alternative treatment for refractory AIED due to better long-term tolerability than cyclophosphamide and fewer side effects than corticosteroids.^[32,33]^ While methotrexate appears effective for treating vestibular symptoms, the hearing response to methotrexate can vary, with 0 to 70% of cases experiencing hearing improvements.^[3–37]^

Azathioprine was found to result in significant hearing improvements in most patients when administered with concomitant prednisone therapy.^[37]^ Noguchi et al suggested that azathioprine combined with oral corticosteroids could be used for hearing losses that preferentially compromise low frequencies and gradual deterioration, such as in patients with hearing loss and Bechet disease.^[38]^ A longitudinal and observational descriptive study showed that the relapse rate of patients treated with azathioprine was 0.52 relapses/year, with 50% relapsing by 9.70 months. In contrast, the relapse rate of patients treated with corticosteroid therapy was 2.01 relapses/year, with patients relapsing at 5.25 months on average (Table 4).^[39]^

**Table 4:** Literature review of pharmacotherapy and treatment responses in autoimmune inner ear disease.

Drug	Year	Author	Type of study	Sample size	Treatment regimen	Hearing loss improvement	Reference
**Corticosteroids**	2005	Niparko et al	Prospective case series	116	Oral prednisone 60 mg/d for 4 wk	69/116 (59.5%)	^[45]^
	2005	Zeitoun et al	Prospective analysis	63	Prednisone 1 mg/kg/d for 7 d (maximum: 60 mg) or methylprednisolone 24 mg, tapered by 4 mg daily for 6 d	28/63 (44%)	^[46]^
**Methotrexate**	1994	Sismanis et al	Prospective open-label	5	Initial dose Of 7.5 mg/wk, increasing to 15 mg/wk (oral)	No significant improvement	^[36]^
	2001	Salley et al	Prospective open-label	50	Initial dose Of 7.5 mg/wk, increasing to 25 mg/wk (oral)	25/47 (53%)	^[47]^
	2001	Matteson et al	Prospective open-label	17	Initial dose of 7.5 mg/wk, increasing to 25 mg/wk in 4 to 8 wk	11/17 (65%)	^[34]^
**Cyclophosphamide**	2001	Lasak et al	Retrospective review	10	100 mg twice a day (oral)	5/10 (50%)	^[31]^
**Azathioprine**				7	100 mg twice a day (oral)	5/7 (71.4%)	
**Rituximab**	2011	Cohen et al	Prospective open-label	7	1000 mg intravenous infusion at baseline and the 15th day	5/7 (71%)	^[48]^
**Anakinra**	2014	Vambutas et al	Prospective open-label	10	100 mg/d subcutaneous injection for 84 d	7/10 (70%)	^[49]^
**Etanercept**	2001	Rahman et al	Retrospective case series	12	25 mg subcutaneous injection twice a week	11/12 (92%)	^[50]^
	2001	Matteson et al	Prospective open-label	23	25 mg subcutaneous injection twice a week for 24 wk	7/23 (30%)	^[51]^
**Infliximab**	2006	van Wijk et al	Nonrandomized prospective pilot study	9	0.3 ml standard solution intratympanic-injected once a week for 4 wk	7/9 (77.8%)	^[52]^
**Plasmapheresis**	1997	Luetje et al	Retrospective chart review	16	One or more times during the active phase of disease	8/16 (50%)	^[53]^

Regarding biological agents, some treatment studies of anakinra and infliximab show higher percentages of hearing improvement.^[40]^ Systemic injection of etanercept exerted its effects through decreasing cochlear inflammation and fibrosis with reduced cochlear infiltrating cell numbers in animal studies,^[41–43]^ and showed its equivalence to corticosteroids in treating induced labyrinthitis.^[44]^

The limitation is that few randomized placebo-controlled trials or treatment protocols demonstrate the efficacy of these alternative therapies in maintaining long-term hearing improvement.^[24]^ In addition, efficacy results are usually controversial due to the lack of consensus on immunopathology and diagnostic criteria of AIED, and the small sample sizes in most studies. In addition, improvement in vestibular symptoms is more significant than hearing symptoms.

The patient in our case report showed only transient responses to oral corticosteroids and intratympanic corticosteroid injections, experiencing 2 relapses at 3 and 14 months. Due to the refractory nature of this disease, immunosuppressant combination therapy was administered, consisting of methotrexate and azathioprine, which resulted in a significant response, leading to the stable return of the ability to detect low- and middle-frequency pure tones (500 and 1000 Hz). The response was defined in line with the recent consensus on hearing improvement as a threshold shift of ≥15 dB at 1 frequency, ≥10 dB at 2 or more consecutive frequencies, or a 12% change in discrimination score within 3 months of commencing the therapy.^[3]^ No side effects were noted in response to methotrexate and azathioprine combination therapy, and the corticosteroid dose was successfully tapered to a maintenance dose of 4 mg/d methylprednisolone.

There are some limitations in this case. First, the diagnosis of AIED is very challenging due to the lack of clear serological tests and biomarkers. We diagnosed AIED based on its typical clinical presentation, disease course, and the exclusion of other possible diseases. Second, although the patient had a good response under methotrexate and azathioprine combination therapy, we can’t well illustrate the special prognostic factors in this patient. Third, we didn’t try other immunosuppressive agents as steroid-sparing agents due to the awareness of possible side effects.

AIED is an uncommon disease. Although corticosteroids are often prescribed as the first-line treatment, treatment responses are variable, and many patients experience a relapse when using corticosteroid monotherapy. We recommend using combination therapy with methotrexate and azathioprine as steroid-sparing agents for patients who are refractory to corticosteroids alone in treatment or have poor tolerability of corticosteroids, as this regimen is well-tolerated with good treatment response.

## Author contributions

**Conceptualization:** Po-Chang Wu.

**Data curation:** Kuan-Hsuan Huang.

**Formal analysis:** Kuan-Hsuan Huang, Hsiao-Ching Lin.

**Validation:** Chia-Der Lin.

Writing – original draft: Hsiao-Ching Lin.

Writing – review & editing: Chia-Der Lin, Po-Chang Wu.
